# Enrichr: interactive and collaborative HTML5 gene list enrichment analysis tool

**DOI:** 10.1186/1471-2105-14-128

**Published:** 2013-04-15

**Authors:** Edward Y Chen, Christopher M Tan, Yan Kou, Qiaonan Duan, Zichen Wang, Gabriela Vaz Meirelles, Neil R Clark, Avi Ma’ayan

**Affiliations:** 1 Department of Pharmacology and Systems Therapeutics, Icahn School of Medicine at Mount Sinai, One Gustave L. Levy Place, Box 1215, New York, NY, 10029, USA; 2 Laboratório Nacional de Biociências (LNBio), Centro Nacional de Pesquisa em Energia e Materiais (CNPEM), Rua Giuseppe Máximo Scolfaro, Campinas, São Paulo, Brazil

## Abstract

**Background:**

System-wide profiling of genes and proteins in mammalian cells produce lists of differentially expressed genes/proteins that need to be further analyzed for their collective functions in order to extract new knowledge. Once unbiased lists of genes or proteins are generated from such experiments, these lists are used as input for computing enrichment with existing lists created from prior knowledge organized into gene-set libraries. While many enrichment analysis tools and gene-set libraries databases have been developed, there is still room for improvement.

**Results:**

Here, we present Enrichr, an integrative web-based and mobile software application that includes new gene-set libraries, an alternative approach to rank enriched terms, and various interactive visualization approaches to display enrichment results using the JavaScript library, Data Driven Documents (D3). The software can also be embedded into any tool that performs gene list analysis. We applied Enrichr to analyze nine cancer cell lines by comparing their enrichment signatures to the enrichment signatures of matched normal tissues. We observed a common pattern of up regulation of the polycomb group PRC2 and enrichment for the histone mark H3K27me3 in many cancer cell lines, as well as alterations in Toll-like receptor and interlukin signaling in K562 cells when compared with normal myeloid CD33+ cells. Such analyses provide global visualization of critical differences between normal tissues and cancer cell lines but can be applied to many other scenarios.

**Conclusions:**

Enrichr is an easy to use intuitive enrichment analysis web-based tool providing various types of visualization summaries of collective functions of gene lists. Enrichr is open source and freely available online at: http://amp.pharm.mssm.edu/Enrichr.

## Background

Recent improvements in our ability to perform genome-wide profiling of DNA, RNA, and protein at lower costs and more accurately further highlight the need for developing tools that can convert such an abundance of data into useful biological, biomedical, and pharmacological knowledge. One of the most powerful methods for analyzing such massive datasets is summarizing the results as lists of differentially expressed genes and then querying such gene lists against prior knowledge gene-set libraries [1,2]. Differentially expressed gene lists can be extracted from RNA-seq or microarray studies; gene lists can be created from genes harboring mutations in cohorts of patients, or gene lists can be putative targets of transcription factors or histone modifications profiled by ChIP-seq. In fact, gene lists can be produced from any relevant experimental method that profiles the entire genome or the proteome. Once unbiased lists of genes or proteins are generated from such experiments, these lists are used as input for computing enrichment with existing lists created from prior knowledge organized into gene-set libraries.

Gene-set libraries are used to organize accumulated knowledge about the function of groups of genes. Each gene-set library is made of a set of related gene lists where each set of genes is associated with a functional term such as a pathway name or a transcription factor that regulates the genes. Creating such gene-set libraries can be achieved by assembling gene sets from diverse contexts. The original method that developed this approach is called gene set enrichment analysis (GSEA), first used to analyze microarray data collected from muscle biopsies of diabetic patients [3]. The authors of this seminal publication developed a statistical test that is based on the Kolmogorov-Smirnov test [4] as well as developed a database of gene-set libraries called MSigDB [5]. Many other gene set enrichment analysis tools have been developed in recent years following the original concept [1]. However, many of such enrichment analysis tools focus on performing enrichment using only the Gene Ontology resource [6]. In addition, enrichment analysis tools most commonly use the Fisher exact test or similar variations of it to compute enrichment [7]. This family of tests has some bias to list size. Besides computing enrichment for input lists of genes, gene-set libraries can be used to build functional association networks [8,9], predict novel functions for genes, and discover distal relationships between biological and pharmacological processes. While many gene-set libraries and tools for performing enrichment analysis already exist, there is a growing need for them and there are more ways to improve and validate gene set enrichment methods. For instance, many useful novel gene set libraries can be created; the performance of the enrichment computation can be improved; and visualization of enrichment results can be done in more intuitive and interactive ways.

Here, we present Enrichr, an integrative web-based and mobile software application that includes many new gene-set libraries, a new approach to rank enriched terms, and powerful interactive visualizations of the results in new ways. Enrichr is delivered as an HTML5 web-based application and also as a mobile app for the iPhone, Android and Blackberry. Users are provided with the ability to share the results with collaborators and export vector graphic figures that display the enrichment results in a publication ready format. We evaluated the ability of Enrichr to rank terms from gene-set libraries by comparing the Fisher exact test to a method we developed which computes the deviation from the expected rank for terms. To evaluate various methods that rank enriched terms, we analyzed lists of differentially expressed genes from studies that measured gene expression after knockdown of transcription factors to see the ranking of the knocked down factors using a transcription-factor/target-gene library [10]. We show that the deviation from the expected rank method ranks more relevant terms higher. We also applied Enrichr to analyze nine cancer cell lines by comparing their enrichment signature patterns to the enrichment signatures of matched normal tissues. Such analysis provides a global visualization of critical regulatory differences between normal tissues and cancer cell lines. In particular, we observed a common pattern of up regulation of the PRC2 polycomb group target genes and enrichment for the histone mark H3K27me3 in many cancer cell lines. The global view of enrichment signature patterns also clearly unravels that Toll-like receptor signaling is turned off in K562 cells when compared to normal CD33+ myeloid cells, whereas interleukin signaling stays intact in both cell types. Overall, Enrichr is an easy to use intuitive enrichment analysis web-based tool providing various types of visualization summaries of collective functions of gene lists.

## Implementation

### Creating the gene-set libraries

Enrichr contains 35 gene-set libraries where some libraries are borrowed from other tools while many other libraries are newly created and only available in Enrichr. The gene-set libraries provided by Enrichr are divided into six categories: transcription, pathways, ontologies, diseases/drugs, cell types and miscellaneous. The following is a description of each library and how it was created:

The transcription category provides six gene-set libraries that attempt to link differentially expressed genes with the transcriptional machinery. These six libraries include the ability to identify transcription factors that are enriched for target genes within the input list using four different options: 1) ChEA [10]; 2) position weight matrices (PWMs) from TRANSFAC [11] and JASPAR [12]; 3) target genes generated from PMWs downloaded from the UCSC genome browser [13]; and 4) transcription factor targets extracted from the ENCODE project [14,15]. In addition, the two other gene-set libraries in the transcription category are gene sets associated with: 5) histone modifications extracted from the Roadmap Epigenomics Project [16]; and 6) microRNAs targets computationally predicted by TargetScan [17].

1. The ChIP-x Enrichment Analysis (ChEA) database [10] is our own resource for storing putative targets for transcription factors extracted from publications that report experiments of profiling transcription factors binding to DNA in mammalian cells. The database is already formatted into a gene-set library where the functional terms are the transcription factors profiled in each study together with the PubMed identifier (PMID) of the paper used to extract the gene. The ChEA gene-set library used in Enrichr is an updated version from the originally published database containing more than twice the entries compared to the originally published version [10].

2. PWMs from TRANSFAC and JASPAR were used to scan the promoters of all human genes in the region −2000 and +500 from the transcription factor start site (TSS). We retained only the 100% matches to the consensus sequences to call an interaction between a factor and target gene. This gene-set library was created for a tool we previously published called Expression2Kinases [18].

3. Transcription factor target genes inferred from PWMs for the human genome were downloaded from the UCSC Genome Browser [13] FTP site which contains many resources for gene and sequence annotations. We converted this file into a gene set library and included it in Enrichr since it produces different results compared with the other method to identify transcription factor/target interactions from PWMs as described above.

4. The ENCODE transcription factor gene-set library is the fourth method to create a transcription factor/target gene set library. We processed the newly published data from the Encyclopedia of DNA Elements (ENCODE) project [14,15]. Using the aligned files for all 646 experiments that profiled transcription factors in mammalian cells, we identified the peaks using the MACS software [19] and then identified the genes targeted by the factors using our own custom processing. We sorted the peaks for each experiment by distance to the transcription factor start site (TSS) and retained the top 2000 target genes for each experiment.

5. The Histone modification gene-set library was created by processing experiments from the NIH Roadmap Epigenomics [20]. Such experiments were conducted using various types of human cell lines types with antibodies targeting over 30 different histone modification marks. ChIP-seq datasets from the Roadmap Epigenomics project deposited to the GEO database were analyzed and converted to gene sets with the use of the software, SICER [21]. Previous studies [22] have indicated that the use of control sample substantially reduces DNA shearing biases and sequencing artifacts; therefore, for each experiment, an input control sample was matched according to the description in GEO. ChIP-seq experiments without matched control input were not included. The resulting gene-set library contains 27 types of histone modifications for 64 human cell lines from various tissue origins.

6. The microRNA gene set library was created by processing data from the TargetScan online database [23] and was borrowed from our previous publication, Lists2Networks [24].

The pathways category includes gene-set libraries from well-known pathway databases such as WikiPathways [25], KEGG [26], BioCarta, and Reactome [27] as well as five gene-set libraries we created from our own resources: kinase enrichment analysis (KEA) [28] for kinases and their known substrates, protein-protein interaction hubs [18], CORUM [29], and complexes from a recent high-throughput IP-MS study [30] as well as a manually assembled gene-set library created from extracting lists of phosphoproteins from SILAC phosphoproteomics publications [31].

1-4.    The pathway associated gene-set libraries were created from each of the above databases by converting members of each pathway from each pathway database to a list of human genes.

5. The Kinase Enrichment Analysis (KEA) gene-set library contains human or mouse kinases and their known substrates collected from literature reports as provided by six kinase-substrate databases: HPRD [32], PhosphoSite [33], PhosphoPoint [34], Phospho.Elm [35], NetworKIN [36], and MINT [37].

6. The protein-protein interaction hubs gene-set library is made from an updated version of a human protein-protein interaction network that we are continually updating and originally published as part of the program, Expression2Kinases [18]. From this network, we extracted the proteins with 120 or more interactions. These proteins are the terms in the library whereas their direct protein interactors are the genes in each gene set.

7–8.    The next two gene-set libraries in the pathway category are protein complexes. The first library was created from a recent study that profiled nuclear complexes in human breast cancer cell lines after applying over 3000 immuno-precipitations followed by mass-spectrometry (IP-MS) experiments using over 1000 different antibodies [30]. The second complexes gene-set library was created from the mammalian complexes database, CORUM [29].

9. The SILAC phosphoproteomics gene set library was created by processing tables from the supporting materials of SILAC phosphoproteomics studies. From each supporting table, we extracted lists of up and down proteins without applying any cutoffs. Protein IDs were converted to mammalian gene IDs when necessary using online gene symbol conversion tools. A total of 84 gene lists were extracted from such studies.

The ontology category contains gene-set libraries created from the three gene ontology trees [6] and from the knockout mouse phenotypes ontology developed by the Jackson Lab from their MGI-MP browser [38]. To create such gene-set libraries, we “cut” the tree at either the third or fourth level and created a gene set from the terms and their associated genes downstream of the cut. The details about creating the Gene Ontology gene-set libraries are provided in our previous publication, Lists2Networks [24].

The disease/drugs category has gene set libraries created from the Connectivity Map database [39], GeneSigDB [40], MSigDB [5], OMIM [41], and VirusMINT [42].

1–2.    The Connectivity Map (CMAP) database [39] contains over 6,000 Affymetrix microarray gene expression experiments where human cancer cell lines were treated with over 1,300 drugs, many of them FDA approved, and changes in expression where measured after six hours. The drugs were always used as a single treatment but varied in concentrations. The CMAP database provides the results in a table where genes are listed in rank order based on their level of differential expression compared to the untreated state. From this table, we extracted the top 100 and bottom 100 differentially expressed genes to create two gene-set libraries, one for the up genes and one for the down genes for each condition. Each set is associated with a drug name and the four digit experiment number from CMAP. This four digit number can be used to locate the concentration, cell-type, and batch.

3. The GeneSigDB gene-set library was borrowed from the GeneSigDB database [40]. The database contains gene lists extracted manually from the supporting tables of thousands of publications; most are from cancer related studies.

4–5.    The OMIM gene-set library was created directly from the NCBI’s OMIM Morbid Map [41]. We removed diseases with only a few genes and merged diseases with similar names because these are likely made of few subtypes of the same disease. In addition, since most diseases have only few genes, we used our tool, Genes2Networks [43], to create the OMIM expanded gene-set library. We entered the disease genes as the seed list and expanded the list by identifying proteins that directly interact with at least two of the disease gene products; in other words, we searched for paths that connect two disease gene products with one intermediate protein, resulting in a sub-network that connects the disease genes with additional proteins/genes. Each sub-network for each disease was converted to a gene set.

6. The VirusMINT gene-set library was created from the VirusMINT database [42], which is made of literature extracted protein-protein interactions between viral proteins and human proteins. Each term in the library represents a virus wherein the genes/proteins in each set are the host proteins that are known to directly interact with all the viral proteins for each virus.

7–8.    The MSigDB computational and MSigDB oncogenic signature gene-set libraries were borrowed from the MSigDB database from categories C4 and C6 [5]. These gene-set libraries contain modules of genes differentially expressed in various cancers.

The cell type category is made of four gene-set libraries: genes highly expressed in human and mouse tissues extracted from the Mouse and Human Gene Atlases [44] and genes highly expressed in cancer cell lines from the Cancer Cell Line Encyclopedia (CCLE) [45] and NCI-60 [46]. The gene-set libraries in this category were all created similarly. The Cancer Cell Line Encyclopedia (CCLE) dataset was derived from the gene-centric RMA-normalized mRNA expression data from the CCLE site. The Human Gene Atlas and Mouse Gene Atlas datasets were derived from averaged GCRMA-normalized mRNA expression data from the BioGPS site. Finally, the Human NCI60 Cell Lines dataset, while also downloaded from the BioGPS site, was raw and not normalized; hence, it was normalized using quantile normalization. The downloaded datasets were all of similar format such that the raw data was in a table with the rows being the genes and the columns being the expression values in the different cells. For each gene, the average and standard deviation of the expression values across all samples were computed. For each gene/term data point, a z-score was calculated based on the row’s average and standard deviation. Duplicate gene probes were merged by selecting the highest absolute z-score. Only genes with an absolute z-score of greater than 3 were selected to be part of a gene set for a particular cell which represents the term.

The miscellaneous category has three gene-set libraries: chromosome location, metabolites, and structural domains. The chromosomal location library is made of human genes belonging to chromosomal segments of the human genome. It is derived from MSigDB [5]. The metabolite library was created from HMDB, a database [47] enlisting metabolites and the genes associated with them. Finally, the structural domains library was created from the PFAM [48] and InterPro [49] databases where the terms are structural domains and the genes/proteins are the genes containing the domains.

### Computing enrichment

Enrichr implements three approaches to compute enrichment. The first one is a standard method implemented within most enrichment analysis tools: the Fisher exact test. This is a proportion test that assumes a binomial distribution and independence for probability of any gene belonging to any set. The second test is a correction to the Fisher exact test that we developed based on intuition. We first compute enrichment using the Fisher exact test for many random input gene lists in order to compute a mean rank and standard deviation from the expected rank for each term in each gene-set library. Then, using a lookup table of expected ranks with their variances, we compute a z-score for deviation from this expected rank, this can be a new corrected score for ranking terms. Alternatively, we combined the p-value computed using the Fisher exact test with the z-score of the deviation from the expected rank by multiplying these two numbers as follows:

(1) $$c=\log\left(p\right)\cdot z$$

Where c is the combined score, p is the p-value computed using the Fisher exact test, and z is the z-score computed by assessing the deviation from the expected rank. Enrichr provides all three options for sorting enriched terms. In the results section, we show how we evaluated the quality of each of these three enrichment methods by examining how the methods rank terms that we know should be highly ranked.

### Visualization of the results on a grid

Enrichr provides various ways to visualize the results from the enrichment analysis. One such method is the visualization of the enriched terms on a grid of squares. Here, all terms from a gene-set library are represented by squares on a grid which is organized based on the terms’ gene content similarity where an area of high similarity is made brighter. To arrange terms on the grid, term-term similarity is first computed using our algorithm, Sets2Networks [9]. For this, the gene-set library is transposed making each gene the set label and the terms the sets for each gene. Sets2Networks then computes the probability for term-term similarity based on a co-occurrence probabilistic calculation. Once an adjacency distance matrix is computed for similarity between all pairs of terms, a simulated annealing process is used to arrange all terms on the dimension-less torodial grid. Dimension-less torodial grid means that the edges of the grid are continuous and connected, forming a torus. The simulated annealing process attempts to maximize the global similarity of terms based on their computed similarity distances as determined by Sets2Networks. The annealing starts with a random arrangement of terms, and then, using the Boltzman distribution, we swap the location of pairs of terms randomly and compute the global fitness of the swap. We run such annealing process until the arrangement converges to a fitness maximum. Once enrichment analysis is computed, the enriched terms are highlighted with higher p-values indicated by a brighter square. The grid can be clicked to toggle between the two alternative views: The alternative view shows all terms on the grid where the enriched terms are highlighted with circles, colored from bright white to gray based on their p-values.

### Computing the significance of clustering of terms on the grid

Once enrichment analysis on the grid is achieved, we compute an index that distinguishes between randomly distributed enriched terms on the grid and terms that significantly cluster. While the continuous case of computing such clustering has a foundation in the literature [50,51], the discrete nature of the grids of terms used in Enrichr has an appreciable effect that makes the computation with the continuous assumption inaccurate. Hence, we implemented a numerical approach to compute such a clustering index with associated probabilities.

### Visualization of the results as a network of terms

Another alternative visualization of the results is to display the enriched terms as a network where the nodes represent the enriched terms and the links represent the gene content similarity among the enriched terms. To make sure the network is sufficiently sparse to avoid clutter and ambiguity, we connected each of the top ten enriched terms to the only other closest enriched term based on gene content similarity. To visualize the network, we slightly modified the force-directed graph example that is a part of the JavaScript library, Data Driven Documents (D3) [52].

### Implementation of the web and mobile applications

Enrichr has two parts: a back end and a front end. The back end is comprised of a Microsoft IIS 6 web server and Apache Tomcat 7 as the Java application server. The back end uses Java servlets to respond to the submissions of gene lists or for processing other data requests from the front end. Apache Maven is used to compile, minify, and aggregate the JavaScript and CSS files for faster web load times, package, and deploy the web app onto the Tomcat server. Conversely, the front end is written primarily in HTML, CSS, JavaScript, and JSP. Enrichr has a user friendly and responsive interface, using AJAX calls to serve JSON response data from the servlet asynchronously for a smoother user experience. The bar graphs, grids, term networks, and color pickers are dynamically generated using the SVG JavaScript library, D3 [52]. The page transitions, sortable tables, hovering over text functions, touch gestures, and other page manipulations are powered by the jQuery JavaScript library. A shared servlet that is used in other projects is used to convert URL-encoded base64 text that represents the SVG figures into downloadable SVG, PNG, or JPG files using the Batik SVG Toolkit from the Apache XML Graphics Project. Enrichr can also be accessed via Android, iOS, and BlackBerry phone apps. All of the phone apps share the mobile framework, Apache Cordova, which allows for the development of cross-platform mobile apps using HTML5, JavaScript, and CSS ensuring that there is no feature decay across the different mobile platforms as well as desktop web platforms. Slight adjustments in Java, Objective C, and JavaScript for Android, iOS, and BlackBerry respectively were necessary to ensure that Enrichr was functional and consistent across these platforms.

### Adding Enrichr as a final step to RNA-seq pipelines

Enrichr's online help contains a Python script that takes as input the output from CuffDiff which is a part of CuffLinks [53]. CuffDiff is a common last step in the analysis of RNA-seq data which finds differentially expressed genes for various comparisons of RNA-seq data. However, the output from CuffDiff is not easy to handle. The python script extracts all the up and down gene lists from the input file, and then using the Python library, Poster, generates links to Enrichr analyses.

## Results and discussion

### The user interface

**Figure 1:**
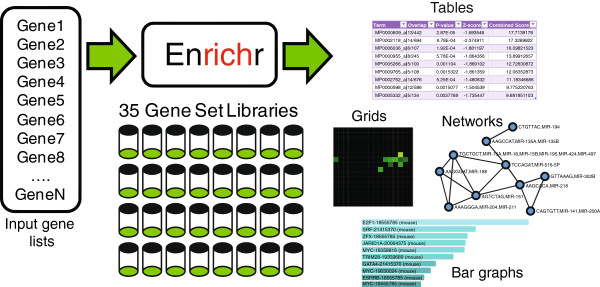
**Enrichr workflow.** Enrichr receives lists of human or mouse genes as input. It uses 35 gene-set libraries to compute enrichment. The enrichment results are interactively displayed as bar graphs, tables, grids of terms with the enriched terms highlighted, and networks of enriched terms.

The user interface of Enrichr starts with a form that enables users to either upload a file containing a list of genes or paste in a list of genes into a text area (Additional file 1: Figure S1). An example is provided to show users the correct format for gene symbols and to enable demo analysis if a gene list is not readily available. Users can optionally enter a brief description of their list, which is useful if they choose to share the analysis with collaborators. After submitting the list for analysis, the user is presented with the results page, which is divided into the six different categories: transcription, pathways, ontologies, disease/drugs, cell types, and miscellaneous. Clicking on the name of the gene-set library expands a box that reveals the enrichment analysis results for that gene-set library. Users are first presented with a bar graph that shows the top 10 enriched terms for the selected gene-set library (Figure 1 and Additional file 2: Figure S2). The bar graph provides a visual representation of how significant each term is based on the overlap with the user’s input list. The longer bars and lighter colored bars mean that the term is more significant. It is possible to export the bar graph as a figure for publication or other form of presentation into three formats: JPEG, SVG and PNG. In addition, the color of the bar graph can be customized using a hexagonal color selection wheel populated with colors that provide the best contrast. There are three methods to compute enrichment and the user can toggle between them by clicking on any bar of the bar graph: Fisher exact test based ranking, rank based ranking, and combined score ranking.

To view the results in a tabular format, the user can switch to the table view tab. The results are presented in an HTML sortable table with various columns showing the enriched terms with the various scores (Figure 1 and Additional file 3: Figure S3). Clicking on the headers allows the user to sort the different columns and a search box is also available if interested in finding the scores for a particular term. Furthermore, the user can export the table to a tab-delimited formatted file that can be opened with software tools such as Excel or any text editor. Within these files, the users can see all terms, their scores, and the overlapping genes with the input genes for each term. The overlapping genes can be seen also by mouse hovering the terms in the table. For most tables, the enriched terms are hyperlinked to external sources that provide more information about the term.

Enrichr also provides a unique visualization of the results on a grid of terms (Figure 1 and Additional file 4: Figure S4). On each grid spot, the terms from a gene-set library are arranged based on their gene content similarity. The enriched terms are highlighted on the grid and color coded based on their level of enrichment, where brighter spots signify more enrichment. Enrichr also provides a measure of clustering of the enriched terms on the grid. The clustering level z-scores and p-values are highlighted in red if the clustering is significant (p-value < 0.1) or displayed in gray if the clustering is not significant. This clustering indicator provides an additional assessment of how related the genes are to each other and how relevant the specific gene-set libraries are for the input list of genes. The observation of one or two clusters on the grid suggests that a gene-set library is relevant to the input list. It also indicates that the terms in the clusters are relevant to the input list. Similar to the bar graph tab, the grid can be customized with the color wheel and exported into the three image formats. Clicking on any spot on the grid toggles between a p-value view and a grid view. The p-value view only highlights the enriched terms leaving all other spots black, while the grid view shows the similarity between terms as bright spots and the enriched terms as circles on top of the grid.

The final visualization option is a network view of the enriched terms (Figure 1 and Additional file 5: Figure S5). The network connects terms that are close to each other on the grid, giving a sense of how the enriched terms are related to each other. The nodes of the network are the enriched terms and they are arranged using a force-based layout. Users have the option to refine this arrangment by dragging the nodes to a desired place. These networks can also be color customized interactively and exported into one of the three image formats.

Enrichr makes it simple to share the analysis results with others. Users can click on the share icon to the right of the description box, resulting in a popup that provides the user with a link to the analysis results that they can copy and paste into an e-mail to send to a collaborator. Users can also create a user account where they can store and organize all their uploaded lists in one place. The user account will enable users to contribute their lists to the community generetaed gene-set library. This will allow other users to query their input lists against user contributed lists.

Enrichr also provides a mechanism to search for functions for specific genes with an auto-complete functionality. The results from the gene function search show all the terms for the gene from all gene-set libraries (Additional file 6: Figure S6). Enrichr is also mobile-friendly such that it supports touch gestures; for example, a simple swipe left and right on the main page switches between the tabs. On the results page, at the top level with no specific enrichment type selected, swipes left and right will navigate between the different enrichment categories. Once the user selects an enrichment type, swipes left and right will navigate between the different visualization types for the current enrichment type.

### Statistics of the gene set libraries

**Figure 2:**
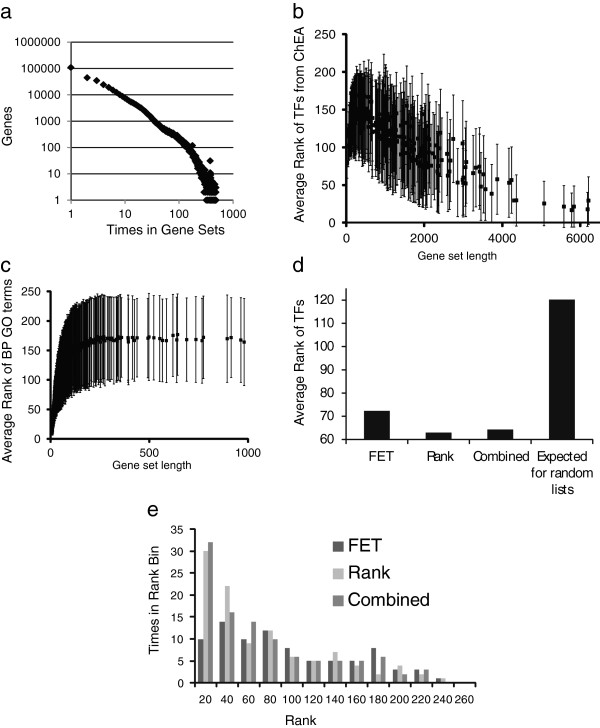
**Validation of enrichment scoring methods.** (**a**) Histogram of overall appearance of genes in gene sets within all the gene-set libraries implemented in Enrichr plotted on a log-log scale; **b-c**) Random gene lists are used to obtain enrichment analysis ranking using the Fisher exact test. Average ranks with their associated standard deviations are plotted against gene list length from the ChEA gene set library **(b)** and the GO Biological Process gene-set library **(c)**; **d-e**) Ranks of specific transcription factors in enrichment analyses using the ChEA gene-set library by the various enrichment analysis scoring methods. Lists of differentially expressed genes after knockdown of the transcription factors with entries in the ChEA gene-set library were used as input; **(d)** Average rank for those factors comparing the three scoring methods; **(e)** histogram of cumulative ranks for the three methods.

Enrichr includes 35 gene-set libraries totaling 31,026 gene-sets that completely cover the human and mouse genome and proteome (Table 1). On average, each gene-set has ~350 genes and there are over six million connections between terms and genes. Further statistics and information of where the gene-set libraries were derived from can be found in the “Dataset Statistics” tab of the Enrichr main page. Histograms of gene frequencies for most gene-set libraries follow a power law, suggesting that some genes are much more common in gene-set libraries than others (Figure 2a). This has an implication for enrichment computations that we did not consider yet in Enrichr. Some genes are more likely to appear in various enrichment analyses more than others, this tendency can stem from various sources including well-studied genes. This research focus bias is in several of the libraries. 

### Evaluation of the enrichment scoring methods

Enrichr computes three types of enrichment scores to assess the significance of overlap between the input list and the gene sets in each gene-set library for ranking a term’s relevance to the input list. These tests are: 1) the Fisher exact test, a test that is implemented in most gene list enrichment analyses programs; 2) a test statistics that we developed which is the z-score of the deviation from the expected rank by the Fisher exact test; and 3) a combined score that multiplies the log of the p-value computed with the Fisher exact test by the z-score computed by our correction to the test. The reason that we devise a correction for the Fisher exact test was because we noticed that some terms always appear on top of the ranked list regardless of the content of the input gene list. This is because the Fisher exact test has a slight bias that affects the ranking of terms solely based on the length of the gene sets in each gene-set library. This can be seen when inputting random gene lists many times and observing the average rank of each term (Figure 2b and 2c). GO terms with few genes are ranked higher if they have short lists and at least one gene from the input list overlaps with the genes associated with the term (Figure 2c). For terms that have enough genes, the rank stabilizes into what is expected for an average rank (slightly above 150 in the plot). For the ChEA enrichment analysis with the Fisher exact test, transcription factors with many targets appear higher more often for random input gene lists (Figure 2b). This is because the ChEA database contain gene IDs that did not match all the genes from our random input lists. Hence, if the gene set library contains “noise,” i.e. gene names that are not standardize, which is very common because gene symbols constantly change and there are many different resources that convert gene/protein IDs to gene symbols, the effect of the Fisher exact test is to give higher rank for terms with longer lists. Since each of the three scoring methods described above produce different ranking for terms, we next evaluated the quality of each of the scoring scheme in an unbiased manner.

**Table 1:** List of gene set libraries ranked by number of terms

		**Gene**	**Mean genes per**
**Gene-set library**	**Terms**	**coverage**	**term**
Down-regulated CMAP	6100	8695	100
Up-regulated CMAP	6100	11251	100
HMDB Metabolites	3906	3729	47.1495
GeneSigDB	2139	23729	126.6947
Human CoR Complexome	1796	10231	158.2778
CORUM	1673	2741	4.6934
Cancer Cell Line Encyclopedia	967	15797	176.2079
GO Biological Process	941	7683	78.4676
MSigDB Computational	858	10061	106.4207
Genome Browser PWMs	615	13362	275.1447
MGI Mammalian Phenotype Top 4	476	10496	201.7101
Kinase Enrichment Analysis KEA	474	4533	36.7089
ENCODE TF ChIP-seq	434	19851	1064.055
GO Molecular Function	402	8469	121.8284
Chromosome Location	386	32740	84.8187
PPI Hub Proteins	385	16487	247.2286
Histone Modifications ChIP-seq	356	21921	1232.129
TRANSFAC/JASPAR PWMs	335	42887	1249.63
Pfam InterPro Domains	311	7588	35.3408
BioCarta Pathways	249	1295	17.6506
ChIP Enrichment Analysis ChEA	240	42574	1455.7
microRNA TargetScan	222	7504	154.6036
GO Cellular Component	205	7325	172.1268
KEGG Pathways	200	4128	48.44
WikiPathways	199	2854	38.8191
MSigDB Oncogenic Signatures	189	11250	165.709
OMIM Expanded	187	2178	88.9198
Mouse Gene Atlas	96	20686	660.1354
NCI-60 Cancer Cell Lines	93	12232	343.3333
OMIM Disease	90	1759	25.0667
VirusMINT	85	851	14.8824
Human Gene Atlas	84	15381	449.7619
SILAC Phosphoproteomics	84	7732	341.869
Reactome Pathways	78	3185	72.5128
MGI Mammalian Phenotype Top 3	71	10406	717.4366

To compare the quality of the rankings of each of these three enrichment analysis methods, we gathered differential gene expression data after knockdown of various transcription factors from 10 experiments extracted from 7 studies (Table 2). Once we have identified lists of statistically significant differentially expressed genes, which are either increased or decreased in expression after the transcription factor knockdown, we examined how the different scoring methods rank putative targets of those factors with the expectation that the knocked-down factors would be highly ranked when applying enrichment analysis with the ChEA gene-set library [10]. This analysis resulted in 104 comparisons of transcription factors ranks because some transcription factors have multiple entries in ChEA. The results show that the second method, the test statistics that corrects the bias from the Fisher exact test, which is the z-score of the deviation from the expected rank, outperforms the Fisher exact test and is comparable with the combined scoring scheme (Figure 2d and 2e). This means that in most cases the method ranks transcription factors higher, based on ChIP-seq data given lists of differentially expressed genes after knockdown of the same transcription factor. The combined scoring scheme is mostly affected by the expected rank test compared with the Fisher exact test, but its overall performance is slightly worse compared to using the expected rank alone. It should be noted that while this analysis shows some advantage to the rank test over the Fisher exact test, more evidence and tests are needed using different gene-set libraries and experimental data to conclusively determine that this rank test is better than the Fisher exact test. However, it is difficult to design such analyses in an unbiased manner and the combination of the ChEA gene-set library coupled with the loss-of-function followed by expression data is the only setting we could devise for such validation so far.

**Table 2:** Rank of entries from the ChEA gene-set library using the three scoring methods implemented in Enrichr given input of lists of up or down regulated genes indentified from studies that profiled gene expression after knockdown or knockout of the same transcription factors

**TF**	**Up/Down**	**PMID**	**Rank p-value**	**Rank z-score**	**Rank combined**
Nanog	Up	16518401	1,4,5,16,	2,4,15,18,22,	1,5,12,16,18,
			28,33,62,144	28,33,116	28,37,117
Nanog	Down	16518401	5,11,14,16,	1,3,4,20,41,	1,6,12,15,18,
			39,58,78,92	54,61,64	56,70,73
Pou5f1	Up	16518401	3,11,12,18,	1,4,12,23,	1,8,14,15,
			27,71,81	33,35,36	21,50,54
Pou5f1	Down	16518401	32,64,78,156,	1,65,92,121,	23,52,90,127,
			176,181,204	160,165,188	171,176,192
Nanog	Up	16767105	3,7,12,18,38,	1,3,11,17,21,	3,5,9,12,25,
			46,56,113	23,26,69	29,36,80
Nanog	Down	16767105	18,28,79,89,	4,17,21,33,44,	23,25,35,48,
			92,102,160,164	83,139,157	60,86,142,186
Pou5f1	Up	16767105	1,9,18,23,31,	2,5,10,20,	1,2,16,20,
			82,120,183	30,34,79	23,55,88
Pou5f1	Down	16767105	25,44,124,166,	47,49,60,131,	43,44,74,134,
			167,180,216	139,169,200	147,153,177
Sox2	Up	16767105	2,10,35,59,61,	11,15,26,36,	3,9,26,44,
			70,121	68,71,103	58,80,123
Sox2	Down	16767105	5,44,50,130,	10,72,85,106,	1,61,82,108,
			139,149,176	110,140,151	116,166,177
Sox2	Up	17515932	2,14,15,41,50,	6,27,30,35,	2,7,24,39,44,
			61,82	44,49,55	45,57
Sox2	Down	17515932	8,19,68,93,117,	6,29,73,95,	4,17,84,103,
			164,216	124,146,210	132,151,168
klf4	Up	18264089	1,27,31,183	6,22,31,199	1,23,31,210
klf4	Down	18264089	61,71,163,200	78,85,190,222	78,79,209,219
Zfp281	Up	18757296	3,24	3,6	3,6
Zfp281	Down	18757296	60,159	63,138	64,147
chd1	Up	19587682	126	106	107
chd1	Down	19587682	231	214	125
Tbx3	Up	20139965	110	96	96
Tbx3	Down	20139965	93	70	76

### Application to obtain a global view of regulatory mechanisms in cancer cell lines and their matching normal tissues

**Figure 3:**
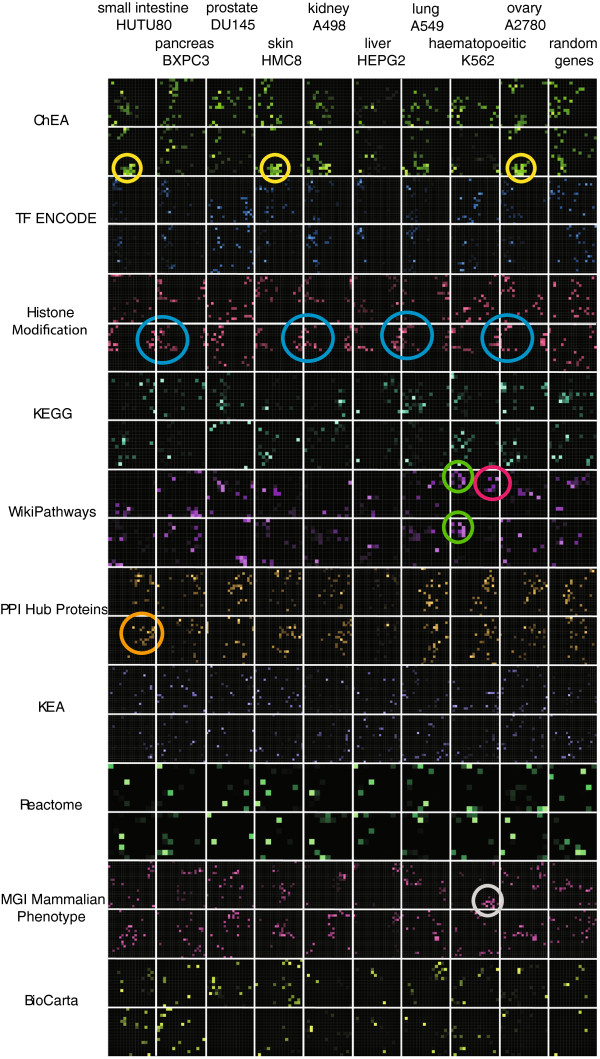
**Global view of signatures created using genes that are highly expressed in cancer cell lines and their matching human tissues.** Enriched terms are highlighted on each grid based on the level of significance using various gene-set libraries, each represented by a different color. Circles are used to highlight specific clusters of enriched terms.

Finally, to demonstrate how Enrichr can be applied globally to obtain a regulatory picture of cancer cell lines and their corresponding normal tissues, we used nine gene sets from the CCLE gene-set library and matching nine gene sets from the Human Gene Atlas library to perform enrichment analysis using ten other gene-set libraries: ChEA, ENCODE TFs, Histone Modifications, KEGG, WikiPathways, PPI Hubs, KEA, Reactome, MGI-MP and Biocarta. We visualize the results using the grid p-value view, coloring each grid with a different color representing the corresponding library (Figure 3). This analysis shows interesting signature patterns: first, we noticed a cluster of transcriptional regulators from ChEA that only appears for the cancer cell lines of ovarian, skin and small intestine cancers. This cluster is composed of the polycomb group complex called PRC2 (highlighted in yellow circles in Figure 3). Next, we saw that, in most of the cancer cell lines, the most enriched terms in the histone modification grids are those associated with H3K27me3 (blue circles in Figure 3). There is direct evidence that the PRC2 polycomb group is responsible for the H3K27me3 specific modification [54], confirming consistency between the ChEA and histone modification enrichment results. Careful examination of the genes for each cancer that overlap with these histone modifications showed that the genes are different for each cancer and are critical tissue specific components. Hence, compared with other cancer cell lines, in these cancer cell lines the PRC2 complex and H3K27me3 modification is used to silence tissue specific genes to help with the dedifferentiation phenotype of cancer cells.

An interesting signature pattern was also present in the WikiPathways grids that compared the enrichment signatures between CD33+ myeloid positive normal hematopoietic cells and K562 cells, which is a cell line often used to study a specific form of leukemia. The two cell lines share a cluster of pathways associated with Interleukin signaling (green circles in Figure 3), but the normal tissue is only enriched with Toll-like receptor signaling cluster, potentially indicating the alteration in signaling in leukemia shutting off this pathway. In addition, the highly expressed genes in the normal hematopoietic cells form a cluster in the MGI-MP grid which are defects in the hematopoietic system when these genes are knocked out in mice (gray circle in Figure 3). Finally, HUTU80 cells, a human duodenum adenocarcinoma cell line, have a cluster in the PPI hubs grid made of the EGFR cell signaling components including EGFR, GRB2, PI3K, and PTPN11 as well as Src signaling including LCK, JAK1 and STAT1, strongly suggesting up-regulation of this pathway in this cancer. Many more interesting clusters and patterns can be extracted from such global view of enrichment signatures and visualization of enriched terms on such grids.

## Conclusions

In conclusion, Enrichr provides access to 35 gene-set libraries with many useful libraries such as those created from ENCODE enlisting many targets for many transcription factors as well as a gene-set library extracted from the NIH Roadmap Epigenomics Project for histone modifications. Other newly created libraries include genes highly expressed in different cell types and tissues; mouse phenotypes from MGI-MP; structural domains; protein-protein hubs; protein complexes; kinase substrates; differentially phosphorylated proteins from SILAC experiments; differentially expressed genes after approved drug perturbations; and virus-host protein interactions. The results from Enrichr are reported in four different ways: table, bar graph, network of enriched terms, and a grid that displays all the terms of a gene-set library while highlighting the enriched terms. Each visual display is easily exportable to vector graphic figures to be incorporated in publications and presentations. Enrichr also has a potentially improved method to compute enrichment, and we demonstrated that this method might be better than the currently widely used Fisher exact test. In addition, we show how figures generated by Enrichr can be used to obtain a global view of cell regulation in cancer by comparing highly expressed genes in cancer cell lines with genes highly expressed in normal matching tissues. Overall, Enrichr is a state-of-the-art gene set enrichment analysis web application. Code snippets are provided to embed Enrichr in any web-site. Enrichr is also available as a mobile app for iPhone, Android and Blackberry.

## Availability and requirements

Enrichr is freely available online at: http://amp.pharm.mssm.edu/Enrichr.

Enrichr requires a browser that supports SVG. Recent versions of Chrome, Firefox, and Opera for Android are recommended. Enrichr only works with Internet Explorer (IE) 9 or higher. In addition, since the stock browsers in Android 2.3.7 (Gingerbread) or below do not support SVG, Enrichr does not work using these browsers.

## Competing interests

The authors declare that they do not have any competing interests.

## Authors’ contributions

AM designed the study, managed the project, wrote the paper, performed various analyses and was responsible for the final submission and revisions of the manuscript. EYC designed the study, implemented the entire application including the design of the web interface, performed various analyses, generated figures and wrote the tutorial. CMT implemented the grid visualization. YK developed the ENCODE and Histone Modification libraries and performed various analyses. QD developed the Python script to analyze CuffDiff output with Enrichr. ZW helped with the development of the code that finds functions for individual genes. GVM developed the SILAC gene set library. NRC developed the statistical method to detect and score clusters on grids. All authors read and approved the final manuscript.

## Supplementary Material

Additional file 1: Figure S1 The initial input interface of Enrichr allows users to cut-and-paste lists of gene symbols or upload a text file containing gene-lists. Click here for file

Additional file 2: Figure S2 Bar graph visualization of the Enrichr results showing the top 10 enriched terms in the ChEA gene-set library. A color wheel is provided to change the bar graph default color. Click here for file

Additional file 3: Figure S3 Table visualization of the Enrichr results showing the top 10 enriched terms in the TRANSFAC and JASPAR PWMs gene-set library. Mouse over events trigger the display of the overlapping genes. The three scoring methods are shown for each term and the complete table can be searched and exported to Excel. Click here for file

Additional file 4: Figure S4 Grid visualization of the Enrichr results showing the top 10 enriched terms in the MGI-MP gene-set library. A color wheel is provided to change the bar graph default color. The z-score and p-value indicate whether the enriched terms are highly clustered on the grid. Click here for file

Additional file 5: Figure S5 Network visualization of the top 10 enriched terms in the Mouse Gene Atlas gene-set library. Enriched terms are connected by their distance on the grid which represents their gene content similarity. Click here for file

Additional file 6: Figure S6 Screenshot from the “Find A Gene” page showing an example for searching annotations for the gene MAPK3. Expanding the ChEA cross shows all gene-sets that contain MAPK3. This means that in those studies MAPK3 was identified as a target gene for the transcription factors. The number next to the transcription factors is the PubMed ID of the study. Click here for file
