# Supplementary figures and images for: Enrichr: interactive and collaborative HTML5 gene list enrichment analysis tool
Source: BMC Bioinformatics. 2013 Apr 15;14:128. doi: 10.1186/1471-2105-14-128 (PMC3637064; doi:10.1186/1471-2105-14-128)

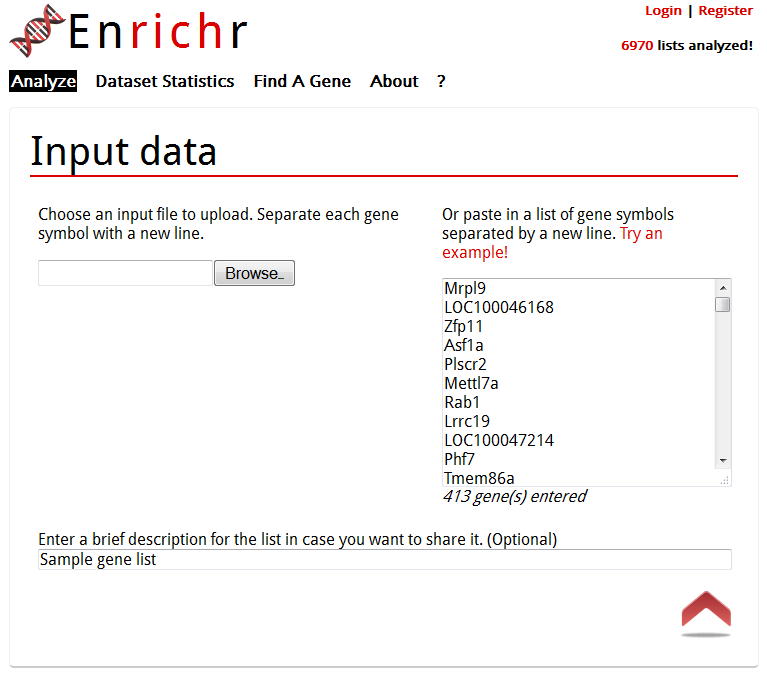

Supplement: Additional file 1: Figure S1 — The initial input interface of Enrichr allows users to cut-and-paste lists of gene symbols or upload a text file containing gene-lists. [file 1471-2105-14-128-S1.png]

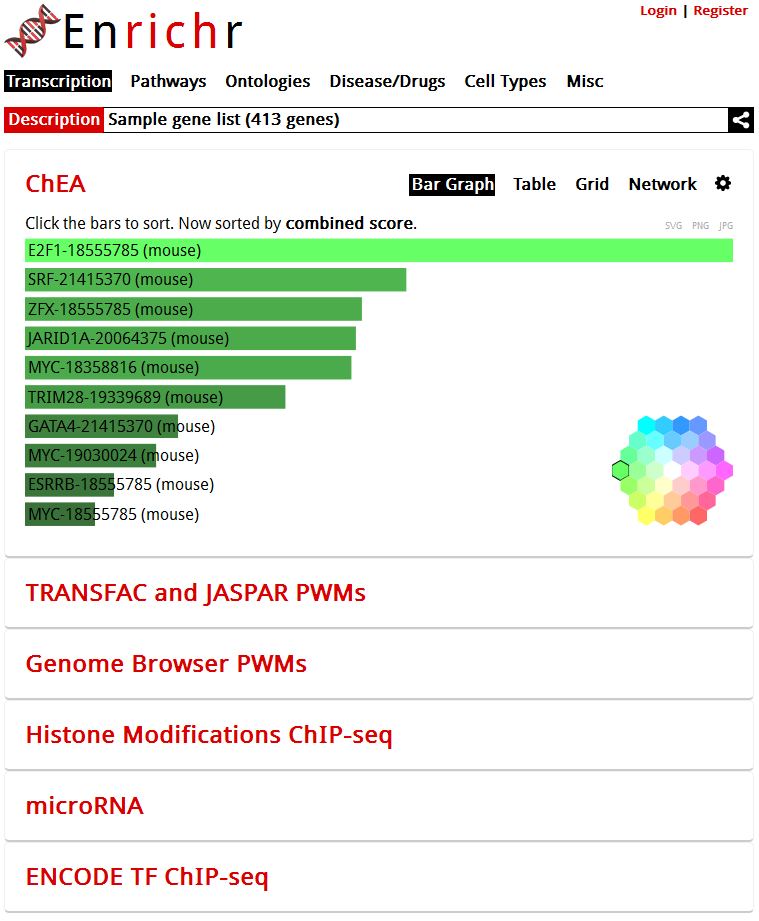

Supplement: Additional file 2: Figure S2 — Bar graph visualization of the Enrichr results showing the top 10 enriched terms in the ChEA gene-set library. A color wheel is provided to change the bar graph default color. [file 1471-2105-14-128-S2.png]

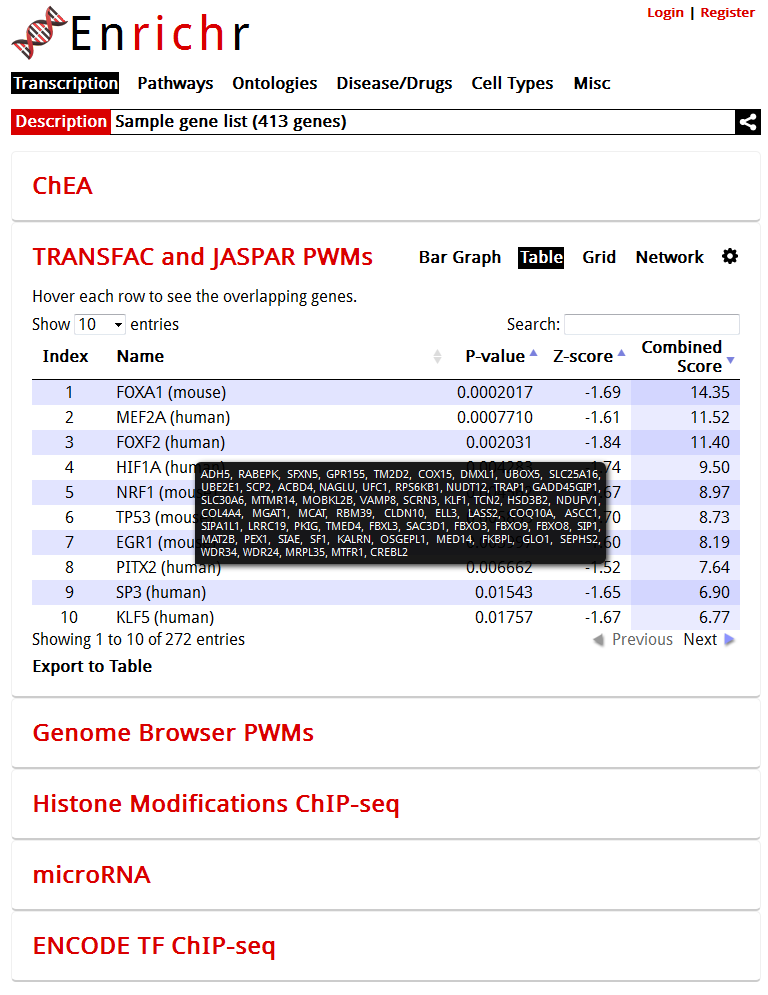

Supplement: Additional file 3: Figure S3 — Table visualization of the Enrichr results showing the top 10 enriched terms in the TRANSFAC and JASPAR PWMs gene-set library. Mouse over events trigger the display of the overlapping genes. The three scoring methods are shown for each term and the complete table can be searched and exported to Excel. [file 1471-2105-14-128-S3.png]

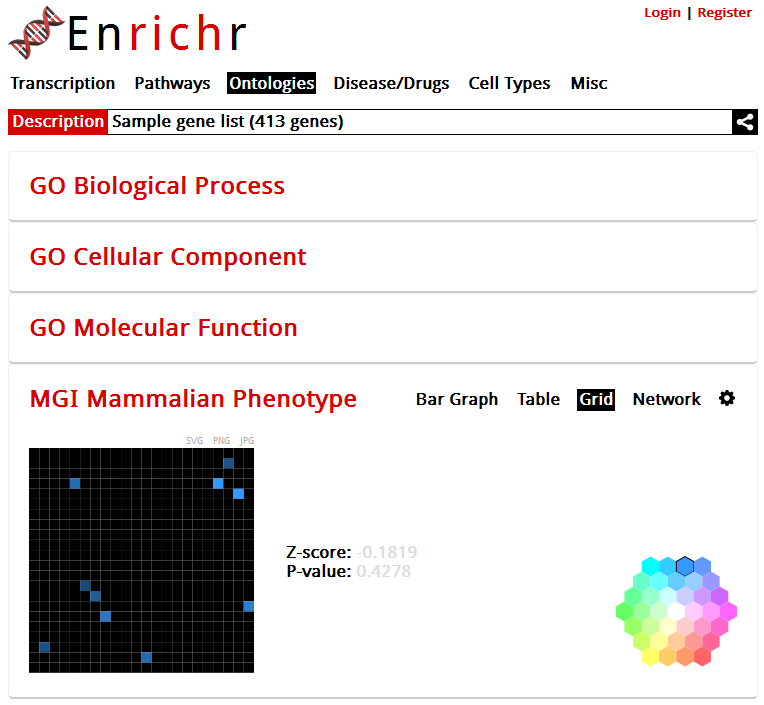

Supplement: Additional file 4: Figure S4 — Grid visualization of the Enrichr results showing the top 10 enriched terms in the MGI-MP gene-set library. A color wheel is provided to change the bar graph default color. The z-score and p-value indicate whether the enriched terms are highly clustered on the grid. [file 1471-2105-14-128-S4.png]

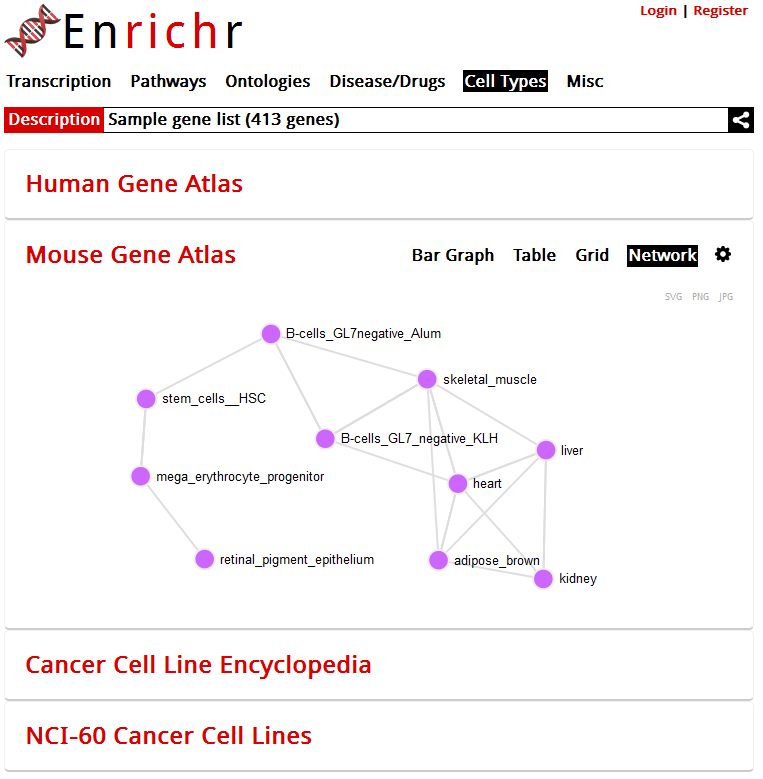

Supplement: Additional file 5: Figure S5 — Network visualization of the top 10 enriched terms in the Mouse Gene Atlas gene-set library. Enriched terms are connected by their distance on the grid which represents their gene content similarity. [file 1471-2105-14-128-S5.png]

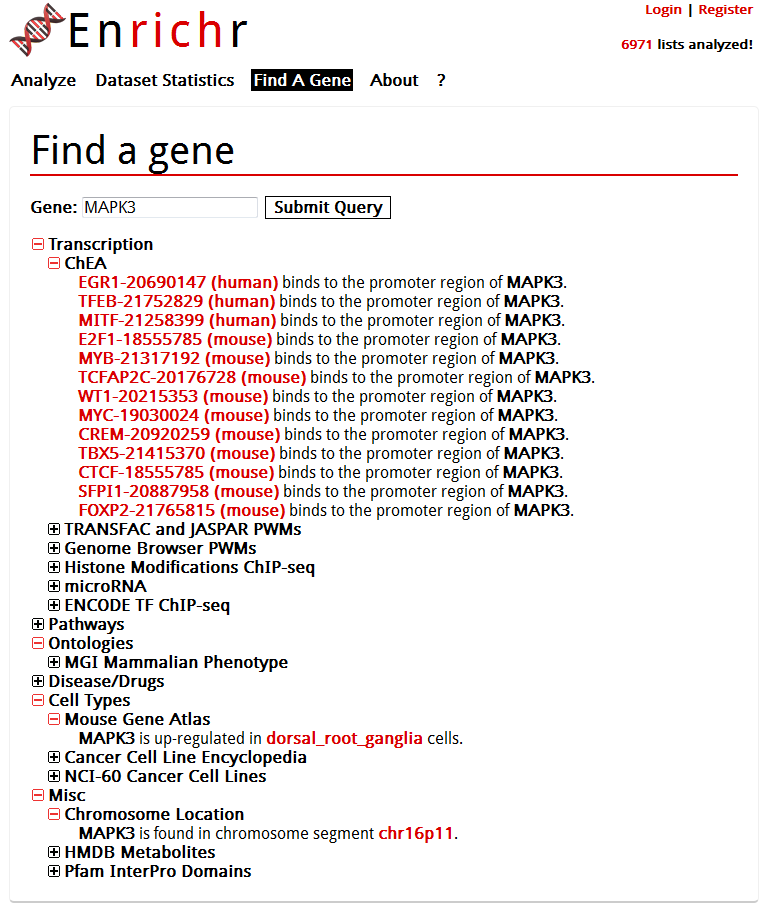

Supplement: Additional file 6: Figure S6 — Screenshot from the “Find A Gene” page showing an example for searching annotations for the gene MAPK3. Expanding the ChEA cross shows all gene-sets that contain MAPK3. This means that in those studies MAPK3 was identified as a target gene for the transcription factors. The number next to the transcription factors is the PubMed ID of the study. [file 1471-2105-14-128-S6.png]
